# Zika vector transmission risk in temperate Australia: a vector competence study

**DOI:** 10.1186/s12985-017-0772-y

**Published:** 2017-06-09

**Authors:** Jean-Bernard Duchemin, Peter T. Mee, Stacey E. Lynch, Ravikiran Vedururu, Lee Trinidad, Prasad Paradkar

**Affiliations:** 1 CSIRO Health and Biosecurity, Australian Animal Health Laboratory, 5 Portarlington Road, Geelong, VIC 3220 Australia; 2 BioScience Research, Agriculture Victoria, AgriBio, The Centre for AgriBioscience, 5 Ring Rd, La Trobe University Campus, Bundoora, VIC 3083 Australia; 3 School of Applied Sciences, RMIT University, Bundoora, VIC 3083 Australia

**Keywords:** Zika virus, Vector competence, *Aedes aegypti*, *Aedes albopictus*, *Culex quinquefasciatus*, Aedes notoscriptus, Australia, Invasive

## Abstract

**Background:**

Zika virus is an emerging pathogen of global importance. It has been responsible for recent outbreaks in the Americas and in the Pacific region. This study assessed five different mosquito species from the temperate climatic zone in Australia and included *Aedes albopictus* as a potentially invasive species.

**Methods:**

Mosquitoes were orally challenged by membrane feeding with Zika virus strain of Cambodia 2010 origin, belonging to the Asian clade. Virus infection and dissemination were assessed by quantitative PCR on midgut and carcass after dissection. Transmission was assessed by determination of cytopathogenic effect of saliva (CPE) on Vero cells, followed by determination of 50% tissue culture infectious dose (TCID_50_) for CPE positive samples. Additionally, the presence of *Wolbachia* endosymbiont infection was assessed by qPCR and standard PCR.

**Results:**

*Culex* mosquitoes were found unable to present Zika virus in saliva, as demonstrated by molecular as well as virological methods. *Aedes aegypti*, was used as a positive control for Zika infection and showed a high level of virus infection, dissemination and transmission. Local *Aedes* species, *Ae. notoscriptus* and, to a lesser degree, *Ae. camptorhynchus* were found to expel virus in their saliva and contained viral nucleic acid within the midgut. Molecular assessment identified low or no dissemination for these species, possibly due to low virus loads. *Ae. albopictus* from Torres Strait islands origin was shown as an efficient vector. *Cx quinquefasciatus* was shown to harbour *Wolbachia* endosymbionts at high prevalence, whilst no *Wolbachia* was found in *Cx annulirostris*. The Australian *Ae. albopictus* population was shown to harbour *Wolbachia* at high frequency.

**Conclusions:**

The risk of local *Aedes* species triggering large Zika epidemics in the southern parts of Australia is low. The potentially invasive *Ae. albopictus* showed high prevalence of virus in the saliva and constitutes a potential threat if this mosquito species becomes established in mainland Australia. Complete risk analysis of Zika transmission in the temperate zone would require an assessment of the impact of temperature on Zika virus replication within local and invasive mosquito species.

## Background

Zika virus was first isolated in Uganda in 1947 from a febrile rhesus monkey. *Aedes* mosquitoes, and primarily *Aedes (Stegomyia) africanus*, was suspected as the main sylvatic vector following direct isolation of Zika virus in 1956 [1]. Subsequent vector competence studies in East [2] and West Africa [3] demonstrated that other members of the *Stegomyia* subgenus showed vector competence such as *Aedes aegypti*, an important vector in Yellow Fever transmission. *Aedes (St.) luteocephalus* has also been incriminated in Nigeria [4], with several Zika virus isolations and a frequent contact with humans. Isolated human cases, serological surveys and virus isolations attested to virus circulation initially in Africa and later in Asia. Virus was isolated from *Ae. aegypti* in Malaysia [5] in 1966 and clinical cases were detected in Indonesia [6] in 1977-1978. However, the authors also suspected *Aedes (St.) albopictus* whose local presence and assumed role in rural dengue transmission made it another possible candidate. More recently, populations of *Ae. aegypti* [7] and *Ae. albopictus* [8] in Singapore have been shown efficacious vectors in the laboratory. Since 2007, Zika virus has successively invaded the Pacific region: Yap island in 2007 [9, 10], then French Polynesia and New Caledonia [11] in 2014. In Yap, the most abundantly collected *Ae. (St.) hensilli*, instead of *Ae. aegypti* was suspected of being responsible for the outbreak, and was shown experimentally capable of infection and dissemination [12]. In French Polynesia, the locally abundant *Aedes (St.) polynesiensis* was found not able to transmit Zika virus [13]. In 2014, Zika virus reached the Americas and *Ae. aegypti* was shown to be a vector, both by molecular detection in field-collected mosquitoes [14] and experimental infection [14–16]. The role of other mosquito species is still under question, especially for *Culex quinquefasciatus*, with conflicting results which either showed it as an efficient experimental vector [14, 17] or not [16, 18, 19].

With a lack of approved vaccines and antivirals, vector control is a key measure for decreasing the public health burden and risk for Zika virus. Identifying vectors and understanding the transmission mechanism is the first step in designing the best suited vector control policy. Despite the presence of Zika virus in the Pacific region (Micronesia and Polynesia), and that, early in the outbreak, travellers returning to Australia have been found to carry the virus [20], no local transmission has been reported. A general consensus identifies the vectors for Zika virus as the same species involved in dengue and chikungunya transmission. Accordingly the global and local risk for Zika virus transmission is set same as dengue and chikungunya risk [21, 22]. In Australia, *Ae. albopictus* is exotic to the mainland and with *Ae. aegypti*’s limited distribution, the dengue risk is limited to the Northern tropical regions. Therefore, local populations of *Ae. aegypti* and potential tropical vectors from Queensland, such as *Ae. (Ochlerotatus) vigilax*, *Ae. (Rampamyia) notoscriptus*, and *Culex. quinquefasciatus*, have been tested for Zika virus vector competence [18]. From this study, *Ae. aegypti* was confirmed as the main suspected vector species, with no other local major or associated species demonstrating virus transmission or considered to play any role. Much of Australia is out of the current dengue risk zone, however the temperate southern zones do harbour dense urban populations and can experience warm summer temperatures. The climatic conditions of these zones is close to those of Southern and Western parts of Europe [23]. Endemic mosquito-borne viruses have been shown to trigger outbreaks in Southern parts of Australia: flaviviruses like Kunjin virus and Murray Valley encephalitis virus, as well as alphaviruses like Ross River virus and Barmah Forest virus. Local species such as *Aedes camptorhynchus*, or populations of *Aedes notoscriptus* and *Culex annulirostris* have been implicated in the circulation of these viruses. Incursion of invasive species, such as *Ae. albopictus* has been detected in the state of Victoria and successfully controlled [24]. *Culex quinquefasciatus* has been well established in Victoria and can be captured during summer. Similar to European researchers who have begun to worry about local Zika transmission [25–27], in order to address the Zika virus transmission risk in the temperate region of Australia, we have performed vector competence experiments on those mosquito species most frequently captured in close association with human populations. As bacterial endosymbiont, *Wolbachia,* infection is known to potentially impact on virus infection in insects [28], we have complemented the competence assessment with *Wolbachia* infection screening for tested mosquito populations. To get a full view of the Zika risk assessment for temperate zones of Australia, we have included *Ae. albopictus* collected from the top northern Torres Strait Island as a potentially invasive species to mainland Australia.

## Methods

### Mosquito sampling

**Fig. 1:**
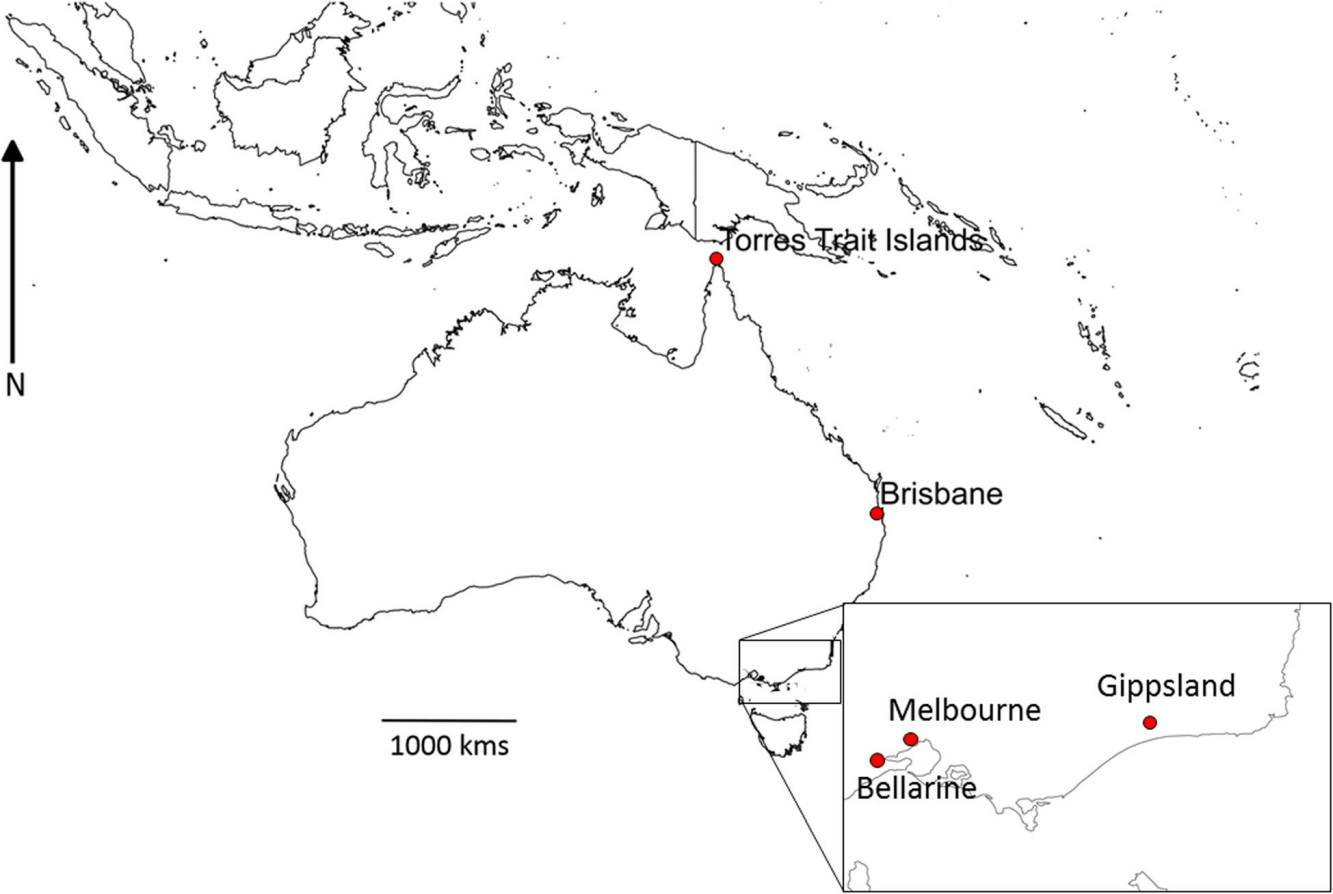
Map showing the origin of the mosquito populations. The Brisbane zone, as cited in the reference numbered 20, is indicated

*- Aedes (Och.) camptorhynchus* samples were collected as larvae in coastal Victorian region of Gippsland (Wellington) (Fig. 1). Following 24 h duration transport to the lab, the larvae were reared in trays with fish food pellets (300 mg/ 100 larvae every 2-3 days) to adulthood. Once at imago stage, they were kept at 25 °C, 65% humidity and under 14:10 day:night photoperiod. Adult mosquitoes were fed 10% sugar solution and starved 24 h before oral virus challenge at 5-8 days old.
*- Aedes (Ram.) notoscriptus* samples were collected as larvae in the Bellarine (Geelong - Highton), and the Melbourne regions (Fig. 1). Conditions of rearing were similar to those of *Ae. camptorhynchus.*
*- Aedes aegypti* colony originated from Cairns, Queensland, Australia. They were reared as described above. The 6th, 7th and 11th generations were used for experimental viral challenge.
*- Aedes albopictus* colony was established from egg batches collected in Hammond Island in the Torres Strait island group, at the top north of Australia, in Dec. 2015. They were reared under the same conditions as described above. The 4th and 9th generations were used for experimental infections.
*- Culex annulirostris* were from a 50 years old colony which originated in Shepparton, Victoria. Rearing principles followed Mc Donald et al. [29]. This colony has been shown to transmit West Nile virus [30].
*- Culex quinquefasciatus* were from a colony established by our laboratory in 2011, from specimens collected in Geelong, Victoria, Australia. Conditions of rearing are similar to *Culex annulirostris*, except oviposition occurred in 10 mL cups instead of petri dishes. Samples used for experimental infection correspond to 30th generation.

The experiments were performed under biosafety level 3 (BSL-3) conditions in the insectary at the Australian Animal Health Laboratory.

### Viral strain

Cambodia 2010 (Genbank KU955593) [31] Zika virus strain was used for *Ae. aegypti*, *Ae. albopictus, Ae. camptorhynchus*, *Ae. notoscriptus*, *Cx quinquefasciatus,* and *Cx annulirostris*. It belongs to the Asian/Pacific/American clade [32] and was passaged once in C6/36 cells and twice in Vero cells before using for mosquito infections.

### Oral challenge

Five to eight days old females were starved the day before being challenged with an infected blood meal (TCID_50_ 10^5.6^/mL) through membrane feeding using chicken blood and skin. Uninfected chicken blood and skin were provided by the Small Animal Facility (Australian Animal Health Laboratory) from chicken bred in the laboratory without any arboviral infection. The procedure was conducted with approval from AAHL Animal Ethics Committee. The blood was spiked with Zika virus just before mosquito blood-feeding. For control, media supernatant was added to the blood before feeding. After one hour, the mosquitoes were anaesthetised with CO_2_ and blood fed females were sorted and kept in a 200 mL cardboard cup at 27.5 °C, 65% humidity and 14:10 day:night photoperiod. The blood-fed specimens were kept for 14 day extrinsic incubation period with 10% sugar solution provided ad libitum.

Sample processing: Specimens were anaesthetised with CO_2_ and saliva was collected as previously described [33], with a slightly modified protocol. Briefly, after removing mosquito’s legs and wings, the proboscis was inserted into a micro-capillary tube containing Foetal Bovine Serum (FBS) and left in place for 20 mins. Micro tubes containing the mix of FBS and expelled saliva were individually stored at -80 °C before virological assessment. Mosquitoes were then dissected in saline phosphate buffer, separating for each individual the midgut, the head with the anterior half of the thorax, and the rest of the carcass, containing ovaries and remains of the exoskeleton.

Virus titre assessment: Saliva testing was performed in two steps: after adding 80 μL of culture media and centrifuging at 2000 g for 3 min, the cytopathogenic effect (CPE) was tested in duplicates on Vero cells (2 × 25 μL) at Day 5 post inoculation. For most samples showing CPE, the TCID_50_ was calculated from the remaining 30 μL using Vero cells as previously described [34].

Molecular testing: After homogenisation of mosquito tissues by bead beating, RNA was extracted using either MagMax (Thermo Fisher) or RNeasy RNA isolation kit (Qiagen, Australia) as manufacturer’s protocol. Ten microliters of RNA was used to prepare cDNA using random hexamers and Superscript-III reverse transcriptase (Thermo Fisher Scientific Inc. Australia) as manufacturer’s protocol. A SYBR Green two step real-time PCR assay was designed for detection of Zika viral partial coding sequence (107 bp) of non-structural protein 5 (NS5) (forward primer: 5′-GAACGAGGATCACTGGATGG-3′, reverse primer: 5′-CTCCTGGTATGCGACTCATC-3′). Screening was conducted using the SYBR™ PreMIX Ex Taq™ II (Takara-Bio Inc., China) and run on a QuantStudio™ 6 Flex Real Time PCR System (Applied Biosystems). Cycling conditions were as follows, 95 °C for 30 s, 40 cycles of 95 °C for 5 s, and 60 °C for 30s, before performing melting curve analysis. The theoretical melting point of a Zika positive sample was 85 °C. However the discriminant melting point with our positive control (Cambodia 2010 strain) was fixed to 80.5 + −0.5 °C. Dilution curve showed positive signal up to the fourth 10-fold dilution, indicative of a concentration of ~100 TCID_50_ / mL. Negative control was a cDNA preparation from Vero cell culture infected with Chikungunya virus. Samples were considered positive if both duplicates had melting temperatures within the positive control range a CT value lower than 40.

#### *Wolbachia* screening

The presence of *Wolbachia* was first assessed for all species except *Ae. albopictus*, using pools of 5 individual cDNA samples obtained from mosquito carcass of the same species. Screening was performed using a SYBR™ Green based quantitative PCR adaptation of the protocol from Mee et al. [35]. Positive pools were retested by conventional PCR, targeting the coding sequences for 16S ribosomal RNA and *Wolbachia* surface protein (wsp) [35].

In a second step, prevalence of *Wolbachia* infection was identified by testing several individuals of each mosquito population, including *Ae. albopictus* with the two conventional 16S and wsp PCR assays.

#### Statistical analysis

Zika infection rate was defined by the number of midguts found positive for viral nucleic acid by qPCR. Similarly, the dissemination rate was calculated by the number of carcasses found positive by qPCR for viral nucleic. Transmission rate was defined by the number of saliva samples showing CPE in Vero cells over the number tested. These different rates were compared by Fisher exact two-tailed test. Average CT and TCID_50_ values were respectively compared by Kruskall-Wallis two-tailed tests.

## Results

Vector competence of six mosquito species for transmission of Zika virus have been assessed.

Our results confirm *Ae. aegypti* as the most efficient vector, with a high rate of midgut infection and dissemination and high virus prevalence and loads in saliva (Table 1)*.*

**Table 1:** Infection, dissemination and transmission rate: calculated from prevalence of viral nucleic acid presence in the dissected midguts and carcasses and prevalence of CPE by saliva samples

Species	Infection rate by qPCR positive midguts (%)	Dissemination rate by qPCR positive carcasses (%)	Transmission rate by CPE (%)
*Aedes aegypti*	40/48 (83%)	39/47 (83%)	33/38 (87%)
*Aedes notoscriptus*	12/35 (34.3%)	2/59 (3.4%)	24/57 (42.1%)
*Aedes camptorhynchus*	5/18 (27.8%)	5/40 (12.5%)	5/37 (13.5%)
*Aedes albopictus*	19/26 (73.1%	19/26 (73.1%)	20/26 (76.9%)
*Culex annulirostris*	0/32 (0%)	0/32 (0%)	0/32 (0%)
*Culex quinquefasciatus*	0/20 (0%)	0/20 (0%)	0/17 (0%)

**Fig. 2:**
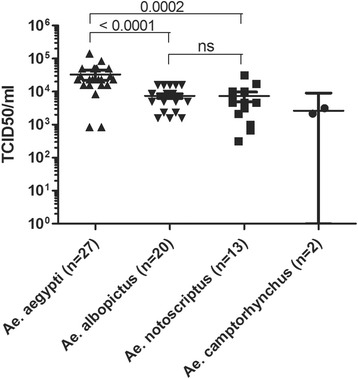
Quantitation of viral load in CPE positive saliva samples. Mosquito samples producing CPE and given a TCID_50_ value were plotted, by species with number of tested samples. Horizontal bars are means with 95%CI. *P*-values of two-tailed Mann Whitney tests are presented (ns = not significant). For *Ae. camptorhynchus*, tests are not applicable due to the low number of values

**Fig. 3:**
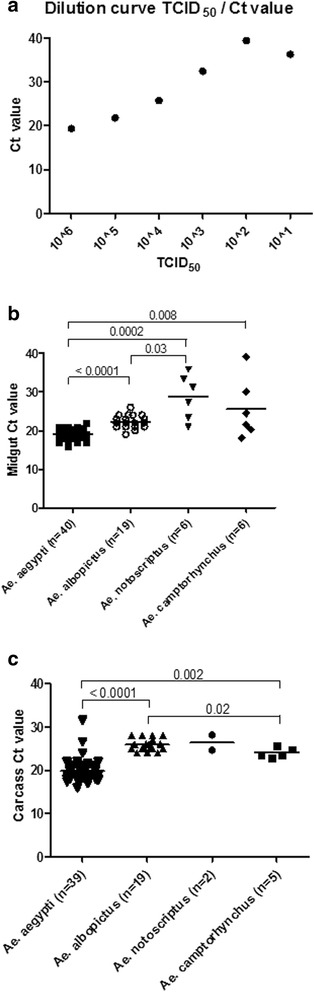
Viral RNA determination in mosquito organs. Real-time RT-PCR was performed on RNA collected from mosquito midgut (**b**) or carcass (**c**) using ZIKV NS5 primers. Horizontal bars as mean with 95% Confidence Interval. For comparison, a dilution curve for TCID_50_ is given in (**a**), where real-time RT-PCR was performed on RNA collected from 10-fold dilutions of Zika virus of known titre. The average Ct value of 3 samples of freshly blood-fed *Ae. camptorhynchus* whole bodies, including the midgut is 30.03; it is equivalent to the viral nucleic acid loading input before any replication, approximatively 10^3.5 of TCID_50_. Significant *p* values are denoted on the graph

*Ae. albopictus* from Australia (Torres Strait Island) was also tested for Zika virus competence and showed high prevalence (>75%) of virus in the saliva at day 14. The TCID_50_ of virus in the saliva of *Ae. aegypti* was found to be significantly higher than *Ae. albopictus* (*p* < 0.0001) and *Ae. notoscriptus* (*p* = 0.0002) (Fig. 2). There was no statistical difference between *Ae. albopictus* and *Ae. notoscriptus* TCID_50_ averages. The CT values for midguts and carcass were significantly lower with *Ae. aegypti* (Fig. 3b).

The results show that two local temperate Aedinae, *Ae. notoscriptus* and *Ae. camptorhynchus*, could be infected by Zika virus and deliver virus in their saliva (Table 1). The molecular screening shows moderate dissemination rates (carcass infection, respectively 3 and 12%), much lower than the infection rates (midgut, respectively 34 and 28%) for both species. We found virus in the saliva of *Ae. notoscriptus* (42%) and *Ae. camptorhynchus* (13.5%) by assessing the cytopathogenic effect of the saliva on Vero cells. The prevalence of virus in the saliva was lower than that of *Ae. aegypti* (87%) (Table 1). To confirm the specificity of the CPE in the saliva as presence of Zika virus, we tested 18 CPE positive wells, corresponding to 12 different mosquito samples (3 *Ae. aegypti* and 9 *Ae. notoscriptus*, randomly chosen), by qPCR. All samples were positive for the presence of Zika virus RNA, with low CT values (average 16.06, with no difference between the two species) (data not shown).

We did not find any evidence of virus in the saliva of the samples of *Cx quinquefasciatus*, and *Cx annulirostris*. In addition, no midgut or carcass sample of any *Culex* species tested positive for viral nucleic acid 14 days post-infection using the Cambodia strain (Table 1).

We have also screened the tested populations for *Wolbachia* presence using molecular assays. *Culex quinquefasciatus* were shown to harbour *Wolbachia*, with 90% prevalence (Table 2), but not *Cx annulirostris. Wolbachia* infection was detected by molecular assay in all of the four *Aedes* species (Table 2). *Aedes albopictus* was the only species found with 100% infection rate, whilst *Ae. aegypti*, *Ae. notoscriptus* and *Ae. camptorhynchus* showed very low prevalence. *Ae. camptorhynchus* showed a positive result by qPCR, however this was not confirmed by conventional PCRs targeting 16S and wsp. Sequencing and blast analysis of the amplicons from both standard PCRs confirmed the presences of *Wolbachia* in *Ae. aegypti, Cx quinquefasciatus* and *Cx pipiens gp.*

**Table 2:** *Wolbachia* infection prevalence for the tested mosquito species

	Pool of 5 mosquitoes			Individual
Species	qPCR (CT value)	16S PCR	Wsp PCR	Prevalence PCR 16S positive
*Aedes aegypti*	+ (15.7)	+	+	1/14
*Aedes albopictus*	NA	+	NA	14/14
*Aedes notoscriptus*	−	−	−	1/15
*Aedes camptorhynchus*	+ (30.8)	−	−	0/15
*Culex quinquefasciatus*	+ (14.3)	+	+	9/10
*Culex annulirostris*	−	−	−	0/15

## Discussion

### *Culex* species

Understanding the importance of *Culex* species, especially Cx *quinquefasciatus,* in Zika virus transmission, with conflicting published data, is crucial for the implementation of vector control policies. Several studies addressing this matter have concluded that *Culex* species may not serve as vectors of Zika virus [16, 18, 19], however two studies found *Cx quinquefasciatus* as competent [14, 17]. Our study did not find any viral RNA or infectious virus in the samples of *Cx quinquefasciatus*. These results confirmed previous experiments performed using tropical Australian *Culex quinquefasciatus* [18], despite the potential differences in genetic background between populations. *Culex quinquefasciatus* was shown to harbour *Wolbachia* endosymbionts, with prevalence of 90% (Table 2). Despite the absence of an obvious relationship between Zika vector capacity and *Wolbachia* natural infection in our results, the screening for associated endosymbionts may be useful in the explanation of discordant results in the different *Culex quinquefasciatus* vector competence studies. Despite being sometimes a major vector for several arboviruses in Australia [36, 37, 38], *Cx annulirostris* was also not found to transmit Zika virus, at 14 days after oral challenge with Cambodia Zika virus strain. The *Cx annulirostris* colony used in this study has a long history and originated in Victoria [29]. Given its origin and the probability of major genetic bottleneck and drift, it may represent a very different population than the natural tropical populations tested by Hall-Mendelin et al. [18]. Additionally, this species has been shown to be composed of several cryptic lineages in Australia [39]. Our study, along with Hall-Mendelin et al. [18], have used *Cx annulirostris* and *Cx quinquefasciatus* from two different zones of Australia for Zika virus vector competence, with negative results obtained regardless of their source location. This places these species in a safe status conferring to no Zika virus transmission, thereby reducing the need for specific vector control.

### *Aedes aegypti*

Contrary to the results obtained from *Culex* species, the data confirm *Ae. aegypti* as the most efficient vector with a high prevalence of midgut infection and dissemination rate detected by molecular screening and high virus prevalence and loads in the saliva. This species is confirmed of prime epidemiological importance and the Australian population, originating from Queensland, is considered as a major vector [18].

### Local temperate Australian *Aedes*

The results of this study demonstrate that two local Aedinae of the temperate zone, *Ae. notoscriptus* and *Ae. camptorhynchus,* can be infected by Zika virus and deliver infectious virus in their saliva. Similar to the results of Hall-Mendelin et al. [18] for *Ae. notoscriptus* and *Aedes vigilax* (a saltmarsh species belonging to the same subgenus as *Ae. camptorhynchus)*, our results showed moderate infection rates and low dissemination rate for these *Aedes* species. However, in contrast to Hall-Mendelin et al., our results showed presence of virus in the saliva of these mosquitoes’ Victorian populations: 42% and 13.5% of *Ae. notoscriptus* and *Ae. camptorhynchus,* respectively. The prevalence of virus in the saliva was lower than that of *Ae. aegypti* (87%) (Table 1). Hall-Medellin et al. reported viral RNA prevalence in saliva at 27% for *Ae. aegypti* at D14. Our results are closer to the 100% transmission results reported by Li et al. [7] who, as we did for saliva, used virological methods (CPE) rather than molecular. Our qPCR molecular test was assessed for relative sensitivity by a dilution curve which showed positive results down to 10^−4^-fold dilution of the initial cDNA from a TCID50 10^6^/ml stock solution, being inconsistent at the 10^−5^ and negative at the 10^−6^ dilution (Fig. 3a). As a further insight of the viral dynamics within the mosquitoes, we tested three whole samples of freshly blood-fed *Ae. camptorhynchus* which gave an average CT value of 30.03 (SD 1.12), as a relative measure of the initial virus load.

The specimens of the population of this mid-sized species were among the larger of tested samples of the different species. The quantity of ingested virus was probably among the higher. The average CT value for *Ae. camptorhynchus* midgut samples is lower than this threshold, indicating a probable local replication after the blood meal. For *Ae. notoscriptus*, the midgut average CT values are similar to the viral input average represented by the whole freshly blood-fed *Ae. camptorhynchus* specimens (Fig. 3b). However, when positive, the carcass of *Ae. notoscriptus* shows lower CT values, indicative of replication (Fig. 3c). Our molecular test could have missed some specimens with low level virus load, either in the midgut or in the carcass. The virological method of virus detection by CPE could be a more robust and reliable method and the most informative indicator of transmission.

Interestingly, following Cornet et al.’s observation, Duschinka et al. [3, 14] proposed a dynamic model of infection in which a transient window time would be best for transmission. This means that mosquitoes would first tolerate virus replication, then would clear them and would not be able to transmit afterwards. Although this is not the current paradigm for virus vector transmission, a recent study [40] has shown that dengue virus would disappear from the saliva of aged *Ae. aegypti*, potentially supporting this temporal transient infection model, even with the “true” vectors.

Beyond the different methodological approaches, the different results between the vector competence assessments performed on two different populations of *Ae. notoscriptus*, from either tropical or temperate zones, could be linked to the vector’s differences in genetic background. Indeed, Endersby et al. [41] hypothesised that *Ae. notoscriptus* could be a complex of cryptic species. Also, screening for *Wolbachia* by conventional PCR on individual carcass (including ovaries) cDNA shows a very low prevalence of *Wolbachia* for the temperate zone population of *Ae. notoscriptus* and absence of positive molecular signal on pools of 5 cDNAs by qPCR. In contrast, a high prevalence of *Wolbachia* natural infection has previously been described in tropical *Ae. notoscriptus* populations either from a colony or field samples collected in Brisbane [42], with this being the same origin as the tropical population tested for Zika virus vector competence [18]. Although natural *Wolbachia* infection did not influence the dengue virus competence of infected *Ae. notoscriptus* populations [42], our study cannot dismiss the possibility of a potential protective effect of natural *Wolbachia* infection on *Ae. notoscriptus* for Zika virus infection and could justify further research. It is to be noted that, despite the distance, *Ae. notoscriptus* has been described as potentially invasive for USA [43]. *Aedes notoscriptus* from Brisbane has been shown to transmit yellow fever virus better than *Ae. aegypti* [44], however *Ae. notoscriptus* of unknown origin failed to develop infections with the same virus [45]. *Aedes notoscriptus* from Brisbane area has been previously shown as a poor vector for the four serotypes of dengue virus [46]. Indeed, populations of this mosquito in its southern distribution range, e.g. the state of Victoria, have not been documented for flavivirus vector competence. No dengue outbreak or documented secondary cases of dengue have been recorded in this region, in the absence of the known dengue vector *Ae. aegypti*. The contact between human populations and *Ae. notoscriptus* has been documented in Queensland but not in temperate regions. *Aedes notoscriptus* has been shown to bite humans 19% of the time for blood meals in residential areas of Brisbane [47]. Its dispersal and survival rates in the same area [48] are similar to *Ae. aegypti* and identify it as a potential vector in urban areas. Although it is known to be a backyard pest in the southern zones, much of its biology remains to be well defined in order to properly assess risk for transmission. The case of *Ae. camptorhynchus* is quite different: its ecology is restricted to coastal areas, or around inland brackish zones [49], with a lower abundance in urban areas [50], with local exceptions in suburb areas around Melbourne, Victoria (SL, pers. Comm.). It has been shown to be an efficient experimental vector for the Alphavirus, Ross River virus [51], and epidemiologically important in the natural transmission of this virus [37]. This includes the possibility of vertical transmission for Ross River and Sindbis viruses [52]. Except this current study, its status as vector for flavivirus is unknown. Similar to *Ae. notoscriptus*, but to a lesser degree, *Ae. camptorhynchus* showed intermediate levels of vector competence for Zika virus. Both are probably not primary vectors, meaning they may be unable to sustain large outbreaks on their own, unlike *Ae. aegypti* in tropical areas, however they could be potential candidates to trigger few secondary cases and reinforce outbreaks in the presence of primary vectors. *Aedes notoscriptus* is known as a backyard pest, due to its abundance and taste for human blood. *Aedes camptorhynchus* has been shown to be responsible for Ross River virus outbreaks, here too displaying a close proximity with humans. However, a big gap in our knowledge remains: the experiments were performed at 27.5 °C. Whilst the state of Victoria can experience very high temperatures and heat waves, on average, summer temperatures are much lower than 25 °C. Performing vector competence experiments at lower temperatures would be useful in establishing a proper risk assessment for local transmission of Zika virus, both by local and potentially invasive species.

### *Aedes albopictus*

Our results demonstrated that *Ae. albopictus,* from Australian Torres Strait island, is a competent vector for Zika virus transmission. This is concordant with Wong et al. [8], who tested *Ae. albopictus* from Singapore and used the dissection of salivary glands and virological method. Beside the methodological approach, with dissection of salivary gland instead of saliva collection, the main differences between the studies are the temperature of rearing (29 °C versus 27.5 °C) and the Zika virus strain (Uganda 1947 strain belonging to the African clade, versus the Cambodia 2010 strain belonging to the Asian clade). *Aedes albopictus* is considered as an invasive species with a large threat potential [53] at a global level, including Europe [27], Africa [54] and Australia. However, variation in results of experimental challenge of vector competence may exist, and could influence greatly the risk model of Zika transmission by *Ae. albopictus* [25]. Studies have reported low (3%) level of experimental transmission, using either molecular or virology method for virus detection in the saliva of infected individuals of European [26] or American [15] *Ae. albopictus* populations. With our results, this demonstrates that distant populations of *Ae albopictus* could show large difference of vector competence. Consequently the risk analysis for this species cannot be carried across different regions, even within comparable climate [23] (Italy and South-Eastern Australia for instance) and needs assessment of local populations of vectors. Interestingly, the tested *Aedes albopictus* population coming from Torres Trait Islands seems to harbour *Wolbachia* endosymbionts. The results do not show them protected from being infected and transmitting Zika virus. However, a more quantitative experimental assessment could answer this question more fully.

## Conclusion

The experimental assessment of vector competence for Australian mosquitoes focusing on the temperate region shows different degrees of risk. *Culex* species are devoid of proof of potential vector competence for Zika virus. Conversely, the potential invader for inland Australia, *Ae. albopictus,* is shown to be a competent vector, with a potential threat at a similar level as found in *Ae. albopictus* population in Singapore and very different from European populations. Two native *Aedes* species show an intermediate level of vector competence, whereby they are probably unable to sustain large outbreaks themselves, but are potential candidates to trigger some secondary cases. A gap in our knowledge exists in lower temperature assessment of vector competence to better mimic local climatic conditions.

## Abbreviations

cDNA: complementary Desoxyribonucleic Acid
CO_2_: Carbon dioxide
CPE: Cytopathogenic effect
CT value: Cycle threshold value
FBS: Foetal Bovine Serum
PCR: Polymerase Chain Reaction
qPCR: real time Polymerase Chain Reaction
RNA: Ribonucleic Acid
TCID_50_: 50% tissue culture infectious dose
Wsp: *Wolbachia* surface protein
